# Long-Distance Sub-Diffraction High-Resolution Imaging Using Sparse Sampling

**DOI:** 10.3390/s20113116

**Published:** 2020-05-31

**Authors:** Duo Wang, Tianjiao Fu, Guoling Bi, Longxu Jin, Xingxiang Zhang

**Affiliations:** 1 Changchun Institute of Optics, Fine Mechanics and Physics, Chinese Academy of Sciences, Changchun 130033, China; wangduo@ciomp.ac.cn (D.W.); futianjiao@ciomp.ac.cn (T.F.); biguoling@ciomp.ac.cn (G.B.); jinlx@ciomp.ac.cn (L.J.); 2 College of Materials Science and Opto-Electronic Technology, University of Chinese Academy of Sciences, Beijing 100049, China

**Keywords:** optical systems, Fourier ptychography, macroscopic imaging, sub-sampling method

## Abstract

How to perform imaging beyond the diffraction limit has always been an essential subject for the research of optical systems. One effective way to achieve this purpose is Fourier ptychography, which has been widely used in microscopic imaging. However, microscopic imaging measurement technology cannot be directly extended to imaging macro objects at long distances. In this paper, a reconstruction algorithm is proposed to solve the need for oversampling low-resolution images, and it is successfully applied to macroscopic imaging. Compared with the traditional FP technology, the proposed sub-sampling method can significantly reduce the number of iterations in reconstruction. Experiments prove that the proposed method can reconstruct low-resolution images captured by the camera and achieve high-resolution imaging of long-range macroscopic objects.

## 1. Introduction

Many visible-light imaging applications are for long-range imaging, such as remote sensing and reconnaissance. Imaging from longer distances usually results in lower spatial resolution [1]. In this case, the resolution of imaging is no longer limited by the magnification of the system itself, but rather by the diffraction of the imaging system. To increase the diffraction limit in optics, we can increase the lens aperture or focal length, but increasing the lens size will make the optical system more cumbersome and affect the performance of the whole system. If we increase the focal length, the cost and system weight will increase rapidly. Therefore, physically increasing the aperture of the lens is not the best solution, and more optical devices must be introduced to compensate for the additional aberrations of the larger aperture. To improve the spatial resolution, super-resolution reconstruction technology can be introduced into computational imaging, and low-resolution images obtained are processed to obtain high-resolution images [2].

Recently, a Fourier ptychography (FP) [3,4,5,6,7] technique is proposed, which uses light waves with different angles to illuminate objects and record all low-resolution diffraction patterns. As a result of the various illumination angles, the spectrum collected in the frequency domain is equivalent to the spectrum obtained in different spatial positions of samples [8]. In traditional imaging, because the aperture of the camera cannot be infinite, the objective lens is equivalent to a low-pass filter, and the spatial spectrum of the object is filtered by the cutoff frequency, resulting in low image resolution. FP technology combines aperture synthesizing [9,10,11] and phase retrieval [12,13,14,15,16]. The high-frequency information of the object is collected by the camera through inclined light wave illumination. Similar to the synthetic aperture, the FP collects information images and integrates them in Fourier space to expand the passband of the optical system, which is equivalent to expanding the equivalent numerical aperture (NA) [17]. Besides, phase imaging is achieved through LED arrays [18]. However, instead of directly recording phase information, FP recovers lost phase information through a phase recovery algorithm. Finally, all low-resolution images are combined with a phase retrieval algorithm to recover high-resolution images. In recent years, this method has provided a new idea for quantitative phase measurement [7], optical aberration correction [19,20], and high-resolution imaging [21]. It has been widely used in quantitative phase microscopy imaging [18], high-speed in vitro live cell imaging [22], super-resolution fluorescence microscopy [23], and other fields.

Recent work shows that FP technology can recover lost phase information by only obtaining the intensity information and bypassing the diffraction limit of the optical system [17]. At present, the application of this technology is often focused on microscopy [24,25,26,27], so the sample must be very thin [3]. The purpose is to obtain the unique different passband mapped to the spectrum of low-resolution images with different angles. To accurately impose the translational spectrum constraints to avoid the thin sample assumption, Dong [28] simply recovered the super-resolution image of the object placed in the far-field by scanning the camera at different positions; Holloway [29,30] used the camera array method of coherent illumination to improve the resolution of imaging the long-distance object.

The biggest problem with FP is that it requires oversampling of the observations, which means that the number of observations must exceed the dimensionality of the problem and sometimes even exceed a large amount. This will cause severe restrictions on the storage and processing of the computer, especially when the target number and the problem dimensions are too large.

To meet the requirements of oversampling, the method adopted in [29] is to overlap the camera array on the same plane continuously, and the overlap of two adjacent cameras exceeds 60%. It is difficult to do in one imaging process, which is challenging to perform in actual production cameras. If the cameras do not overlap, their experimental results are not ideal.

Based on Reference [29], we intend to use very few observation samples, especially to use samples that are much smaller than the problem dimension to solve the problem of oversampling in FP. Therefore, we propose a sub-sampling method, which is applied to the existing FP technology to realize the imaging of macroscopic objects at a long distance.

## 2. Principle

### 2.1. Optical Path Setting

**Figure 1:**
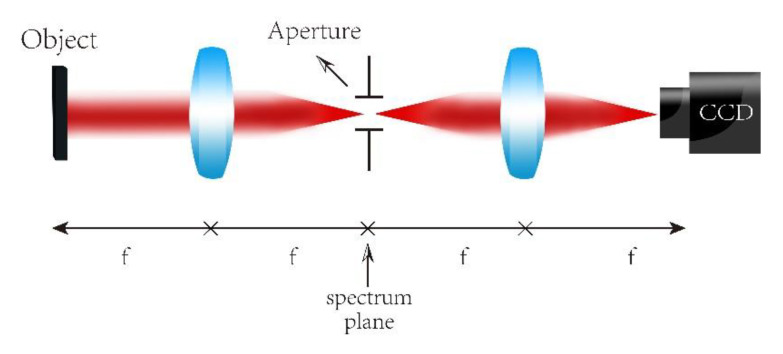
Classic 4f optical system. Two lenses with focal length f are required.

Typical FP technology is based on a 4f system and its optical path structure are shown in Figure 1. Suppose the amplitude function of the object is g(x,y); after passing through lens 1, the expression on the Fourier spectrum surface is $G(f_{x},f_{y})$. If a light pass hole is set on the spectrum surface of the 4f system, and the aperture size is $d(f_{x},f_{y})$, then the image of $G^{\prime }(f_{x},f_{y})$ will selectively transmit to the charge-coupled device (CCD)by moving the circular hole. For each position of the pinhole denoted as i, then the intensity of the image obtained from the object $I_{i}$ is $I_{i}={\left|ℱ\left[d_{i}(f_{x},f_{y})\cdot G^{\prime }(f_{x},f_{y})\right]\right|}^{2}$, where ℱ is the Fourier transform operator. By processing these captured low-resolution images in the frequency domain, a high-resolution image of the object is finally achieved.

**Figure 2:**
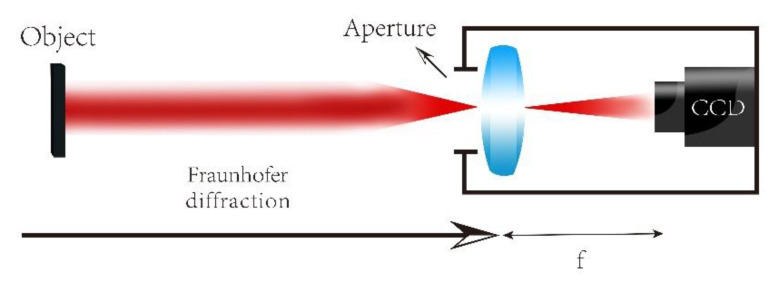
Path of the 4f system simplified by far-field diffraction.

The physical method of Fourier transform operation for a plane-transmitting object is to realize its Fraunhofer diffraction [31]. To observe the far-field diffraction pattern of an object at a close distance, it is often necessary to use a traditional optical element, namely a lens. In this way, the role of the optical lens is equivalent to the Fourier transform of the object. For long-distance imaging systems, there is no need to add a lens [28,32]. Therefore, the optical path structure in Figure 1 is changed to Figure 2, and the aperture of the camera lens replaces the small hole in Figure 1 as an actual diaphragm for far-field imaging. Then, only moving the camera can realize the effect of synthesizing a broader passband in Fourier space, thereby avoiding the diffraction limit of the optical camera’s resolution.

### 2.2. Imaging Model

The computational reconstruction process of macro Fourier ptychography imaging requires the intensity information of the object recorded by the image sensor to restore the complete spectrum information of the object. This is mathematically equivalent to the phase recovery problem. To ensure that the problem is solvable, the Fourier ptychography reconstruction algorithm uses low-resolution intensity constraints and overlap rate constraints to reconstruct the object’s spectrum.

**Figure 3:**
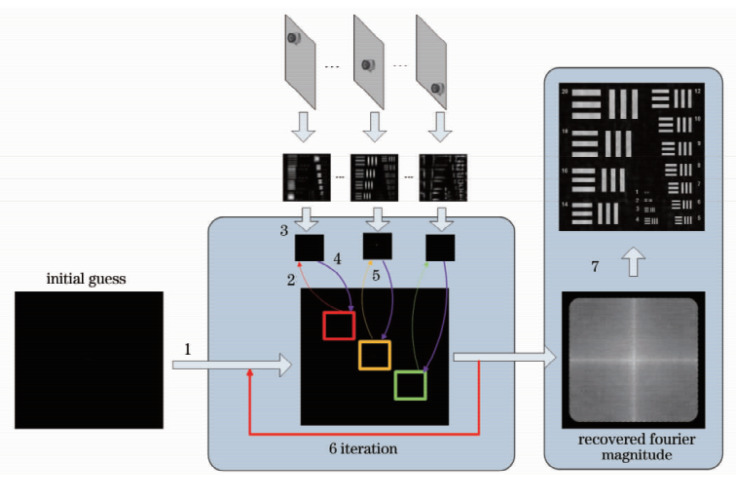
Entire reconstruction process of Fourier ptychography.

The basic principle of the phase recovery algorithm used is to iteratively update the low-resolution image to obtain the synthesized spectrum and obtain the high-resolution image. Figure 3 shows the reconstruction process of Fourier ptychography.

The reconstruction steps are as follows:

(1) Assumed sample initial spectrum: $\varphi_{0}\left(x,y\right)$
(2) Set the aperture function $D\left(x-x_{i},y-y_{i}\right)$ represents the aperture of the lens, i represents the camera at the i position, multiplied by the object spectrum to obtain the spectrum obtained by the aperture: $\varphi_{i}\left(x,y\right)=\varphi_{0}\left(x,y\right)\cdot D\left(x-x_{i},y-y_{i}\right)$.
(3) Inverse Fourier transform of the intercepted spectrum to the spatial domain: $\tilde{\varphi_{i}}={ℱ}^{-1}\left(\varphi_{i}\right)$; replace the corresponding position amplitude with the intensity of the image collected by the detector but retain the phase: ${\tilde{\varphi_{i}}}^{\prime }=\sqrt{I_{i}}\frac{\tilde{\varphi_{i}}}{\left|\tilde{\varphi_{i}}\right|}$.
(4) Transform the ${\tilde{\varphi_{i}}}^{\prime }$ Fourier transform to the frequency domain to obtain $\varphi_{i}{}^{\prime }=ℱ\left({\tilde{\varphi_{i}}}^{\prime }\right)$ and update the spectrum: $\phi_{i}=\phi_{0}+\eta \frac{D_{i}{}^{*}\left|D_{i}\right|}{\left({\left|D_{i}\right|}^{2}+\gamma \right)\max\left(\left|D_{i}\right|\right)}\times \left(\varphi_{i}{}^{\prime }-\varphi_{0}\right)$. In the formula, η is the forgetting factor, which determines the ratio between the previous iteration value and the next iteration value, which affects the iterative convergence rate; γ is the adjustment factor, and the purpose is to ensure that the denominator is not 0.
(5) Update the spectrum at i+1 positions until the spectrum at all positions has been updated. At this point, complete one iteration.
(6) Continue the iteration until the preset number of iterations t is reached or the iteration error is less than the threshold.
(7) Get the final synthesized spectrum $\phi_{t}{}^{\prime }$, and take the square of the inverse Fourier to transform to obtain the reconstructed image: $I_{t}=\left|{ℱ}^{-1}\left(\phi_{t}{}^{\prime }\right)\right|$.

## 3. Reconstruction Algorithm

In this section, a new phase retrieval algorithm is proposed: it is obtained by integrating the alternating minimization phase retrieval (AMPR) [13,33] with the sparse phase retrieval by truncating the amplitude flow (SPARTA) [34]. The method described in [13,33] requires a sufficiently accurate initial signal at the beginning. The method described in [34] can be closer to the real signal after each iteration, but the trouble is that the samples need to be updated each time again. The integrated method can avoid the shortcomings of the respective algorithms.

Suppose $y_{i}=\left|Ax\right|$, where i=1,2…m, our purpose is to measure the matrix $A=\left[a_{1}{,a}_{2}\ldots a_{m}\right]$ to recover the complex vector x, y is the amplitude information recorded by the detector, and x belongs to the complex field. In other words, given y and A to recover x, the recovery process is called phase recovery. Then, the optical path structure of Figure 2 can be expressed in mathematical form: $A_{i}=R_{i}{ℱ}^{-1}D_{i}ℱ$, where $R_{i}$ is the sub-sampling operator, which can retain the measured amplitude value, $D_{i}$ is the pupil at the i th position, and it can intercept different bandwidths in the Fourier domain. Assuming that the signal is sparse when only the amplitude value y is given to calculate the signal x, we use the least square criterion, and then the problem is transformed into the known sparsity k to solve the signal x from the equation without phase. Then, we use the estimation of the signal as a solution to the non-convex optimization: $\mathrm{minimize}\left(x\right):\frac{1}{2m}{\sum_{i=1}^{m}\left[\left|A\left(x\right)\right|-y_{i}\right]}^{2}$ where m is the measurements. To introduce the above scheme into the FP framework, we rewrite the imaging expression $I_{i}={\left|ℱ\left[d_{i}(f_{x},f_{y})\cdot G^{\prime }(f_{x},f_{y})\right]\right|}^{2}$ to $y_{i}=\left|A_{i}x\right|$, where y represents the amplitude of the low-resolution image obtained by the camera, i.e., $y_{i}=\sqrt{I_{i}}$, $A_{i}$ represents the transform matrix of the inverse Fourier transform, and x corresponds to the high-resolution spectrum. Then, the problem becomes a standard phase recovery problem. If the real phase $A^{T}x$ is P, then the problem can be expressed as: $Py=A^{T}x$, where P is the diagonal matrix of phases.

**Figure 4:**
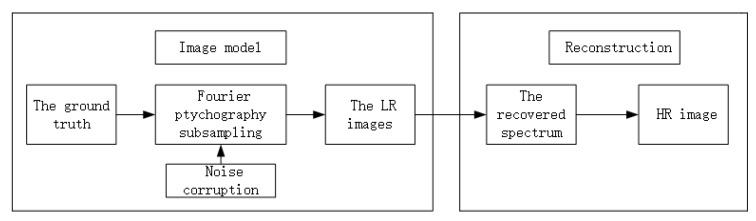
Framework for implementing long-range macro imaging.

Since we cannot measure P directly, we have to find a suitable method for phase retrieval. Considering that the traditional FP algorithm is not ideal for the measurement or phase recovery of a sparse signal, and its reconstruction effect is not ideal, we intend to apply the theory of compression perception to remote high-resolution imaging: a sparse image in the transform domain is recovered with a lower sampling rate. This can effectively reduce the storage of devices and the complexity and time cost of sampling. The structural framework of the algorithm is shown in Figure 4. Therefore, we combine the method of phase retrieval by alternating minimization [33] and sparse phase retrieval by truncated amplitude flow [34] to find the phase information P that we want to obtain, so as to realize the imaging of long-distance macro objects. For the specific implementation steps, see Algorithm 1.

The main problem of the algorithm AMPR is that the initial value is relatively weak, and the solution may not converge. It is required to give a good enough initial estimate. Therefore, we used the root-mean-square measurement to replace the non-singular value for the initial guess of the signal in the initial stage. After solving the initialization problem to obtain a simple initial estimate, we then use an alternating minimization algorithm to improve this estimate. The specific method is to estimate the initial phase according to $A_{i}=R_{i}{ℱ}^{-1}D_{i}ℱ$ in any iteration and assign the obtained initial phase to the intensity information collected by the detector. Next, we use a sparse recovery algorithm to obtain an estimate of the next signal.

We combined and improved the two methods of phase retrieval using alternating minimization and sparse phase retrieval by a truncated amplitude stream and applied it to long-range high-resolution imaging. In the following, the effectiveness of the algorithm is proved through experiments.

In order to make the code concise, we have made the following definitions and descriptions: the signal power $\psi^{2}$ is well approximated by the average power in the measurements; $x^{0}$ will give us a good initial approximation of the real signal x; $A^{T}$ is the transpose of the vector-matrix A; we also define $\mathrm{Ph}(z)\overset{\mathrm{def}}{=}\frac{z}{\left|z\right|}$, and ${ℋ}_{k}(u)$ sets all entries of u to zero except for the k-ones of largest magnitudes.
**Algorithm 1** Long-distance sub-diffraction high-resolution imaging for sparse sampling**Input**: Sampling matrix *A_i_*,captured LR images *y_i_***Parameters**: Maximum number of iterations *t*, step size *μ*, sparsity level *k***Set**: Set $\overset{\wedge }{S}$ to include indices corresponding to the *k*-largest instances:                                                      $\psi^{2}=\frac{1}{m}\overset{m}{\underset{i=1}{\sum }}y_{i}^{2}$                             Compute the principal eigenvectorz of matrix:                                                 $Y=\frac{1}{m}\overset{m}{\underset{i=1}{\sum }}y_{i}^{2}A_{i,\overset{\wedge }{S}}A^{T}{}_{i,\overset{\wedge }{S}}$**Initialize**:                                                      $x^{0}\leftarrow \sqrt{\frac{1}{m}\overset{m}{\underset{i=1}{\sum }}y_{i}^{2}}$                                                 {Initial approximation}                                                                 t=0**Loop**:                                                               t←t+1                                                 $\varphi^{t+1}\leftarrow \mathrm{diag}\left(\mathrm{Ph}\left(A^{T}x^{t}\right)\right)$                                                 {Initial approximation}                             $x^{t+1}\leftarrow {ℋ}_{k}\left(x^{t}-\frac{u}{m}\underset{i=0}{\sum }\left(A^{T}x^{t}-\;\varphi_{i}\frac{A^{T}x^{t}}{\left|A^{T}x^{t}\right|}A\right)\right)$                                             {obtain the next signal estimate}**Output**: Recovered spectrum $x^{t}$.

## 4. Experimental Verification

### 4.1. Experimental Design

**Figure 5:**
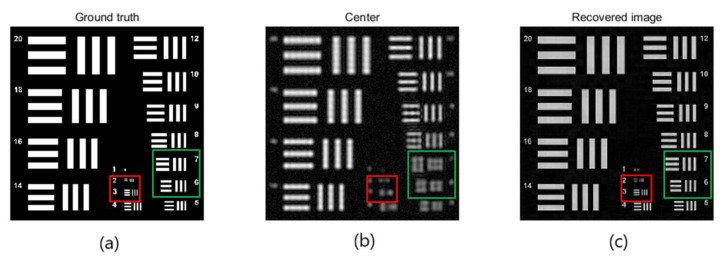
Effect of long-range images is a reconstruction using Fourier ptychography (FP) technology. (**a**) Ground truth: we simulate imaging a 64 × 64 mm resolution target 50 m away using the sensor with a pixel pitch of 2 µm. The width of a bar in group 20 is 25 mm. (**b**) Center image: The target is observed using a lens with a focal length of 800 mm and an aperture of 18 mm. The aperture is scanned over a 17 × 17 grid (61% overlap) creating a synthetic aperture of 130.2 mm where the synthetic aperture ratio (SAR) is 7.24. We can see it has lost at least 14 pixels of its features due to the low-pass filtering of the aperture. (**c**) Recovered image: Using FP technology to restore high-frequency information, the resolution of the image can be improved, at least to restore the characteristics of 2 pixels.

For optical imaging systems, the imaging of distant targets will result in a lower spatial resolution due to the limitation of the lens aperture. To avoid too low resolution, usually use a large-diameter lens. This will increase the weight of the lens and increase the corresponding cost. Therefore, in the design experiment, we intend to artificially synthesize a larger aperture. In the experimental simulation, we use a 512 × 512 USAF resolution chart, as shown in Figure 5a. The chart contains line pairs with widths ranging from 20 pixels to 1 pixel. Suppose the illumination wavelength is 550 nm, the focal length is 800 mm, and the aperture is 18 mm. The resolution chart itself is 64 mm × 64 mm and is located 50 meters away from the camera. For simulation experiments, we assume that the pixel width of the image sensor is 2 µm. It should be noted that in the simulation experiment, we only regard the blur of the image as the limitation of the resolution of the remote image.

In our first simulation experiment, we capture a 17 × 17 grid of images with 61% overlap between neighboring images. However, due to the effect of low-pass filtering, high-frequency information is lost. With the help of the lens scanning on the spectrum surface, we artificially synthesized a larger aperture. We define a concept: synthetic aperture ratio (SAR), which is the ratio between the synthetic aperture diameter and lens aperture diameter. In the first experiment, the aperture is 18 mm; the value of the synthetic aperture is 130.32 mm, SAR = 7.24. Figure 5b shows the image obtained from the center has lost at least 14 pixels of its features due to the low-pass filtering of the aperture. If we use FP technology, the resolution of the image is much improved; features as small as 2 pixels can be recovered, as shown in Figure 5c. The next thing we need to do is to reconstruct high-definition images based on a small amount of observation data.

We will divide several experiments to prove the effectiveness of the algorithm. First, we will prove the probability that the algorithm can recover in the case of sparse; then, we will build a sub-sampling method to recover the signal in the case of sparse constraints; finally, we test the case that the image can be recovered in the case of reducing the amount of aperture overlap.

### 4.2. Success Probability of Algorithm Recovery

**Figure 6:**
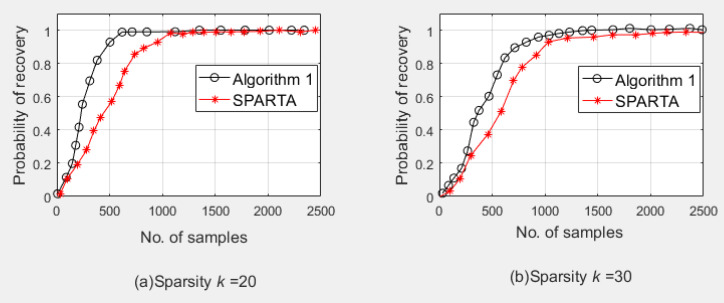
Phase shift diagram, signal length n=2000: (**a**) Sparsity k=20; (**b**) Sparsity k=30.

In the first experiment, the phase shift was used to evaluate the algorithm’s success probability for signal recovery. A signal length of n=2000 and a sparsity of k=20 and 30 were set. The probability test results recovered by Algorithm 1 are shown in Figure 6. We can see that as the signal sparsity increases, a phase shift to the right will occur. At the same time, compared with the SPARTA algorithm, we can observe that as k increases, SPARTA moves more obviously to the right than the phase change of Algorithm 1, indicating that Algorithm 1 requires lower sample complexity.

### 4.3. Subsampling

#### 4.3.1. Pixel Uniform Sub-Sampling

A sub-sampling template was designed, and the elements on the template obeyed the binomial distribution. The function of the template is that there are only 1 and 0 operations in the matrix operation, 1 is maintained in the required area, and the discarded area becomes 0. That is, only the pixels corresponding to 1 remain on the pupil. Sub-sampling is performed in N cameras that obtain intensity information, and the number of values is No.=p∗(nN), where p represents the proportion of sampled samples, and N represents the total number of camera lenses.

The United States Air Force (USAF) resolution chart is selected as the sparse experimental object. The USAF resolution chart can better observe the details of each algorithm in the restored image, so as to compare the experimental results.

The parameters selected by the algorithm are a $n=512^{2}\left(512\times 512\right)$ image (the USAF resolution chart) as the ground truth image, this image has an only amplitude and no phase information; the camera array consists of N=289(17×17) cameras; the camera aperture size is 75 pixels; the overlap rate between the two cameras is set to 0.7; Gaussian noise is added to each image (SNR = 30 dB); the number of iterations in the phase recovery loop is set to 30; and the proportion of sub-sampling accounts for 0.3 of the original measurement data.

**Figure 7:**
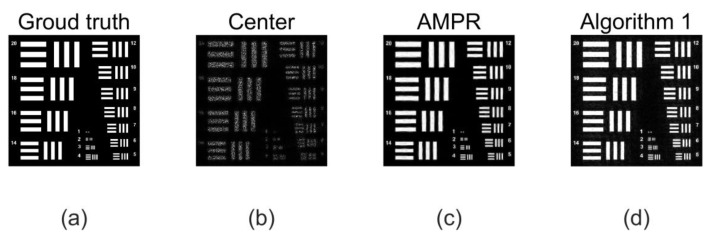
Experimental results using pixel uniform sub-sampling (**a**) Ground truth: we simulate imaging a 64 × 64 mm resolution target 50 m away using the sensor with a pixel pitch of 2 µm. (**b**) Center image: Structural Similarity Index (SSIM) = 0.2866. The target is observed using a lens with a focal length of 800 mm and an aperture of 18 mm. The aperture is scanned over a 17 × 17 grid (70% overlap) creating a synthetic aperture of 104.4 mm synthetic aperture ratio (SAR) is 5.8, (**c**) Alternating minimization phase retrieval (AMPR): SSIM = 0.3968. The output of phase retrieval is obtained using the AMPR algorithm. (**d**) Algorithm 1, SSIM = 0.7932. The output of phase retrieval is obtained using Algorithm 1. We can see that Algorithm 1 is improved from 0.3968 to 0.7932 compared to the algorithm AMPR.

**Figure 8:**
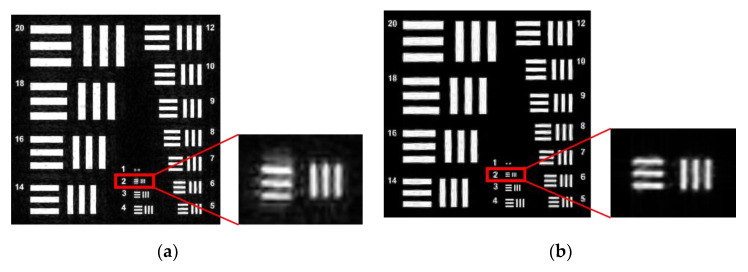
Partial enlargement of Figure 6. (**a**) Partially magnify the results of AMPR recovery; (**b**) Partially magnify the results of Algorithm 1 recovery. When we use the sub-sampling method, we can see that the blur of the algorithm AMPR in the details is significantly higher than Algorithm 1 from the detail image of the image reconstruction effect. The details of the recovered image show that Algorithm 1 improves the quality of image reconstruction.

To achieve this, the $R_{i}$ template was designed for this purpose. For the implementation of Algorithm 1, the sparsity k=0.25n is set, and the Structural Similarity Index (SSIM) is used as the evaluation index. On the basis of FP, Algorithm 1 is used for sparse phase retrieval, and the experimental results are compared with the method of alternating minimization phase retrieval (AMPR) in Reference [29]. The experimental comparison results of the recovered image are shown in Figure 7. In order to be able to see the local details, (c) and (d) in Figure 7 are enlarged, and the effect is shown in Figure 8.

**Figure 9:**
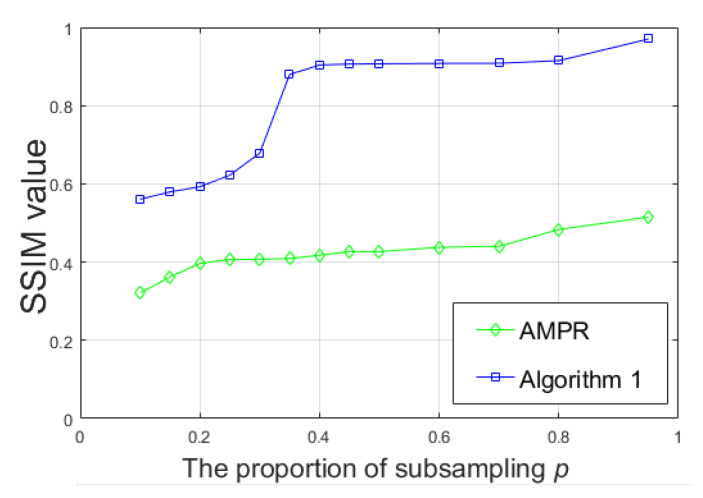
In SSIM at different sampling rates.

At the same time, we studied the change of the sub-sampling ratio and also used SSIM as the evaluation standard to evaluate the effect of image restoration. The experimental results are shown in Figure 9. We can observe that no matter what the proportion p of the sample is, the effect of Algorithm 1 on the image is always better than that of AMPR from Figure 9.

#### 4.3.2. Using Camera Sub-Sampling

**Figure 10:**
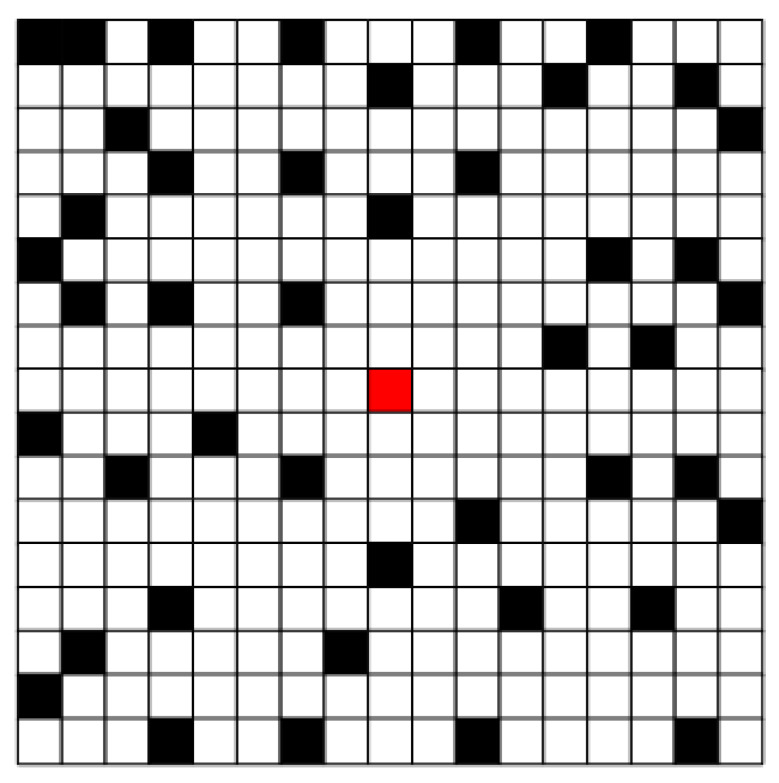
Schematic diagram of camera switch status. The center red is always on by default, the black part is off, and the white part is on.

**Figure 11:**
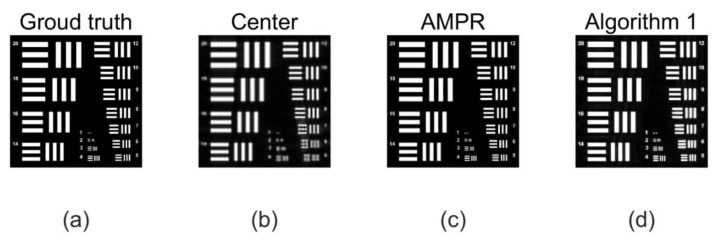
Experimental results using camera sub-sampling, 50% cameras are active (**a**) Ground truth: we simulate imaging a 64 × 64 mm resolution target 50 m away using the sensor with a pixel pitch of 2 µm. (**b**) Center image: SSIM = 0.3198. The image is acquired by the intermediate camera. The aperture is scanned over a 17 × 17 grid (70% overlap) creating a synthetic aperture of 104.4 mm synthetic aperture ratio (SAR) is 5.8, (**c**) AMPR: SSIM = 0.4384. The output of phase retrieval is obtained using the AMPR algorithm. (**d**) Algorithm 1, SSIM = 0.8899. The output of phase retrieval is obtained using Algorithm 1. We can see that Algorithm 1 is improved from 0.4384 to 0.8899 compared to the algorithm AMPR.

**Figure 12:**
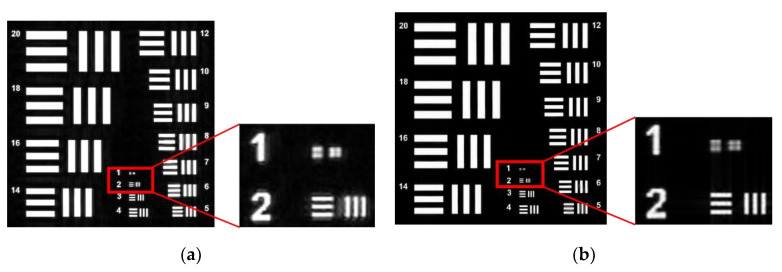
Partial enlargement of Figure 11: (**a**) Partially magnify the results of AMPR recovery; (**b**) Partially magnify the results of Algorithm 1 recovery. When we use the camera sub-sampling method, we can see that around the two line pairs, there is a blur around the algorithm AMPR, but Algorithm 1 is basically no blur and can be well resolved. This shows that the quality of Algorithm 1 in image reconstruction is better than the algorithm AMPR.

The camera sub-sampling method is different from the pixel uniform sub-sampling method. The specific implementation method is to randomly leave some cameras on and leave the others off. We also design the template $R_{i}$. In the case of this sub-sampling, the state of the camera is shown in Figure 10, where the center camera is turned on by default. Out of a total of 289 cameras, 146 cameras are on and 143 cameras are off. About half of the cameras are active. Algorithm 1 executes the same parameters as in Section 4.3.1. The experimental comparison results of the recovered image are shown in Figure 11. To be able to see the local details, (c) and (d) in Figure 11 is enlarged, and the effect is shown in Figure 12.

### 4.4. Reduce the Case of Aperture Overlap

**Figure 13:**
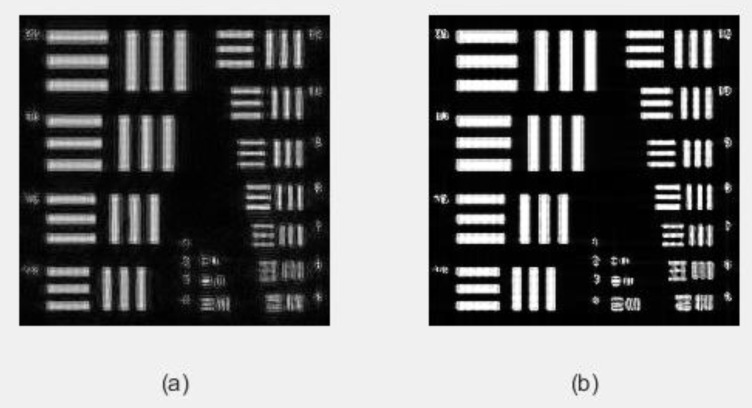
The overlap rate was reduced from 0.7 to 0.2. (**a**) The effect of reconstruction using the AMPR algorithm, SSIM = 0.2143; (**b**) The effect of reconstruction using Algorithm 1, SSIM = 0.5941.

The defect in Reference [29] is mentioned in Section 1: a large area overlap of camera array is required. For common phase retrieval methods, oversampling is necessary. If the overlap rate is less than 0.6, the reconstruction effect is very unsatisfactory. However, if the overlap rate is too large, it means that more cameras overlap on the same plane, which can not be operated in practice. To this end, sparse constraints are added to the algorithm to achieve the high-quality restoration of the target. In the experiment, the amount of overlap between the two cameras was reduced from 0.7 to 0.2 (p=1). Other condition parameters were not changed. The experimental comparison results of the recovered images are shown in Figure 13 when the overlap rate drops to 0.2.The experimental results show that even if the amount of overlap is reduced, Algorithm 1 has a better reconstruction effect compared with AMPR.

## 5. Conclusions

In this paper, we propose and prove the reconstruction method of the macro object image for pixel super-resolution. In the proposed method, we avoid the oversampling problem of traditional FP technology. Compared with the original application of FP technology for macro object imaging, we greatly reduce the need to keep the camera fixed on the platform and then obtain the image in a sequence for tens of minutes. On the one hand, it solves the problem of too long time caused by oversampling, so as to improve the imaging speed; on the other hand, it also enhances the SSIM of the restored image, and accordingly only recovers more details of the real image from the low-resolution image obtained by the camera. From the experimental results, no matter whether in the visual effect or quantitative index, our method uses less low-resolution images, consumes less time, and still can get a better-reconstructed image. Therefore, it is expected that our approach can be applied to many other problems that require phase retrieval.
